# Cognitive profile of kidney transplant patients and impact of deceased vs. living donor transplantation

**DOI:** 10.1007/s40620-024-02004-8

**Published:** 2024-07-11

**Authors:** Johanna Marie Doerr, Martin Juenemann, Anna Becker, Christian Nahrgang, Lucy Rainer, Juliane Liese, Andreas Hecker, Martin Wolter, Rolf Weimer, Hristos Karakizlis

**Affiliations:** 1 Department of Neurology, Justus-Liebig-University of Giessen, Giessen, Germany; 2 Department of Internal Medicine, Justus-Liebig-University of Giessen, Giessen, Germany; 3 Department of General, Visceral, Thoracic, and Transplant Surgery, Justus-Liebig-University of Giessen, Giessen, Germany

**Keywords:** Kidney transplantation, Cognitive performance, Cognitive profile, Chronic kidney disease

## Abstract

**Background:**

It is important to learn more about the prevalence, severity and characteristics (i.e., which cognitive abilities are especially affected) of cognitive impairment in kidney transplant patients. Furthermore, the impact of living vs. deceased donor renal transplantation on cognitive outcome in this patient group needs further studies.

**Methods:**

Fifty-nine patients (43 men, age 55 ± 13 years) who received a deceased donor or living donor kidney transplant, completed a comprehensive neuropsychological test assessment. Neuropsychological tests explored the cognitive domains of verbal and visual memory, attention, and executive functions.

**Results:**

Fifteen percent  of the patients had mild, 25% moderate, and 15% severe cognitive impairment. The level of domain-specific cognitive deficit differed between verbal memory, attention, and executive functions (*χ*^*2*^*(2)* = 7.11*, p* = 0.029*)*. On average, patients showed the highest deficit in executive functions, and the lowest deficit in verbal memory. Patients who received a kidney graft from a deceased donor were more likely to have a cognitive impairment than those who received a kidney graft from a living donor (*OR* = 3.03, 95% CI [0.99,9.32], Wald *χ*^*2*^_*(1)*_ = 3.74, *p* = 0.053). This effect was independent of time on dialysis as well as of creatinine levels, or creatinine clearance.

**Conclusions:**

Our results show that in kidney transplant patients with cognitive impairment, the cognitive domain of executive functions is the most affected one. This might be detrimental for quality of life. The fact that patients who received living donor kidneys seem to do better in terms of cognition than patients with deceased donor kidneys deserves more attention in future research.

**Graphical abstract:**

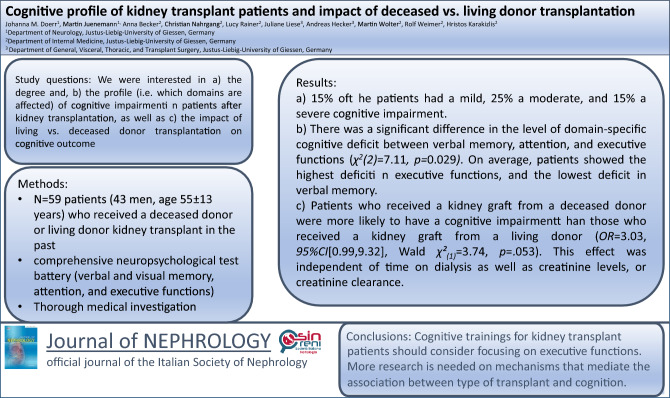

## Introduction

Kidney transplantation is the preferred therapy for patients with end-stage kidney disease (ESKD). Although an association of cognitive impairment with chronic kidney disease (CKD) has been reported over the last decade in up to 80% of patients [1–3], there are limited data on cognitive performance in patients after kidney transplantation. It has been demonstrated that patients after kidney transplantation showed better cognitive performance than dialysis patients [4]. Additionally, there is some evidence from prospective studies that shows an improvement in cognitive performance after kidney transplantation [5]. However, kidney transplant recipients are still significantly more likely to be affected by cognitive impairment or dementia than age-matched comparison groups [3, 6]. The cognitive profile (i.e., which cognitive domains are particularly affected by a decline) has not yet been studied in great detail in kidney transplant patients. This, however, is of great importance, because it may improve treatment options and give valuable information on possible pathways of cognitive decline. As patients with ESKD share common cardiovascular risk factors with patients who suffer from (small vessel) subcortical vascular dementia, the profile of cognitive impairment is suspected to be similar to this patient group [7]. This would mean that the patients show greater cognitive difficulties in concentration, as well as in executive functions (e.g. cognitive flexibility and planning) than in memory [8]. One of our own previous investigations in dialysis patients with ESKD showed that, indeed, the cognitive domain of executive functions is particularly affected [2]. There are also few studies that investigated cognition of kidney transplant recipients using neuropsychological tests covering various cognitive domains (for an overview see [9]). However, none of these studies directly compared performance between cognitive domains within patients.

In our opinion, this important topic deserves more attention, as cognitive impairment can cause a high individual loss of quality of life and early restriction of self-determination and of therapy adherence [10]. Cognitive decline, especially so in the executive domain, may also limit the ability to overview the advantages and disadvantages of various renal replacement therapies, and ultimately may lead to more frequent hospitalizations [6] or even higher risk for graft loss and higher mortality [11]. Early detection and thorough description of cognitive impairment in kidney transplant recipients is therefore of paramount significance to take preventive action, assess illness-related impairment, and to avoid misunderstandings during medical care [12].

Also, besides well-known, transplant-unrelated, risk factors of cognitive decline such as older age, depression, or cardiovascular factors, there is further need to investigate possible transplant-related factors facilitating cognitive decline. In particular, the type of transplantation (living-donor kidney vs. deceased-donor kidney transplantation) could be of interest. Previous research showed that patients who received kidney grafts from living donors do better in terms of graft survival, and ultimately mortality, than patients who received an organ from deceased donors [13]. As kidney function was found to be associated with cognitive impairment [14], there might also be a difference in cognitive impairment between recipients after living kidney donation and those with deceased-donor kidney grafts. Recipients of kidney grafts from living donors usually spent less time on dialysis before transplantation, and dialysis per se has been suggested to cause cognitive decline [15]. A possible influence of the type of transplantation could therefore be explained by time on dialysis before transplantation.

In summary, the goals of our study were a) to derive a profile of cognitive function in kidney transplant recipients using validated neuropsychological tests, and b) investigate the association of the type of transplantation with cognitive impairment in this patient group. Our hypotheses were:

1. A significant proportion of patients who received a kidney transplant in the past show at least a mild cognitive impairment (i.e., a deficit in at least one cognitive domain; definition see below)
2. When comparing the cognitive domains of verbal and visual memory, attention, and executive functions, the domains of executive function and attention will be more affected than the other domains.
3. There is an association between type of transplant (living donor vs. deceased donor) and being at least mildly cognitively impaired. This association is mediated by time on dialysis pre transplantation or current kidney function (creatinine levels).

## Methods

### Study design and enrollment

For the current analysis, we used data assessed as part of a larger study investigating cognitive outcomes and related factors in kidney transplant patients. A detailed description of our study design including the cognitive tests used has been previously published [16]. Here, we only report measures relevant to the current research questions in patients who had already received a kidney graft at the time of inclusion in the study.

We conducted a prospective single-center cohort study at the University Hospital Giessen and Marburg, Giessen, Germany. It complies with the Declaration of Helsinki, has been approved by the ethics committee of the Justus Liebig University Giessen (ref 195/20) and was registered with the German clinical Trials register (ID: DRKS00029164). Written informed consent was signed by all patients as well as the investigator prior to the patient’s enrollment. For the current analyses, we include results of cognitive testing at baseline.

### Participants

We included patients who had already undergone kidney transplantation and were in outpatient follow-up. Inclusion criteria comprised being fluent in German and at least 18 years of age. Individuals under the legal supervision of a caregiver and with preexisting major psychiatric disorders were excluded from the study. At baseline, patients performed cognitive tests, and completed several questionnaires assessing demographic and medical variables [16]. Furthermore, patients underwent a medical examination and blood sampling.

### The cognitive test battery

We assessed the cognitive domains of verbal and visual memory (both delayed recall), attention, and executive function using the following validated cognitive tests: The Verbal Learning and Memory Test (VLMT) [17], the Rey-Osterrieth Complex Figure Test (ROCFT) [18], the Trail Making Test (TMT) A and B [19], and the subtest digit span of the Wechsler Memory Scale revised (WMS-R) [20]. Patients also underwent the Mini-Mental Status Examination (MMSE) [21] as a cognitive screening. For a description of the tests in greater detail see [16]. Except for the Mini-Mental Status Examination, raw scores were transformed into z-scores (mean (M) = 0, standard deviation (SD) = 1) using norms which adjust for age, and, whenever available, sex as well as years of education.

We considered the mean z-score of the following sub-tests as representing the performance in the following cognitive domains:

- Verbal memory (delayed recall): VLMT free word recall after 20–30 min, and VLMT word recognition
- Visual memory (delayed recall): ROCFT free recall after 20–30 min
- Attention: TMT A, and digit span forward (WMS-R)
- Execute functions: TMT B, and digit span backward (WMS-R).

If patients performed between 1.5 and 1.99 SD below the norm sample in at least one sub-test of a domain, they were considered to have a mild deficit in this domain. A performance of ≤ 2.0 SD below the norm in at least one sub-test of a domain was considered a severe deficit in this domain. Patients’ overall cognitive impairment at baseline was then stratified using an algorithm adopted by Murray and colleagues [1] based on the Mayo criteria for mild cognitive impairment (MCI) [22] and the Diagnostic and Statistical Manual, fifth Edition, criteria for major neurocognitive impairment as approximate guidelines [23]:

1. *Unimpaired patients*: no deficit in any domain.
2. *Mildly impaired patients*: mild deficits in only one domain.
3. *Moderately impaired patients:* mild deficits in two domains or a severe deficit in one domain.
4. *Severely impaired patients*: severe deficits in at least two domains.

### Statistical analysis

Statistical analyses were performed using SPSS version 29.0 (IBM). For the first research question, we used descriptive analysis of the data. The influence of possibly confounding variables on degree of cognitive impairment (no, mild, moderate, severe) was investigated using ordinal logistic regressions with degree of cognitive impairment as outcome variable.

For the assessment of the second research question (differences between deficit severity in cognitive domains), we calculated a Friedman test to account for the within-person design in which the variables (i.e., domain-specific deficit severity) are nonparametric. This analysis was only conducted in the sub-sample of patients with at least a mild cognitive impairment, and only included the cognitive domains of verbal memory, attention, and executive function due to missing variance in visual memory performance (only two participants had deficits in this domain: one mild, one severe). For post-hoc analyses of significant effects, we calculated Friedman pairwise comparisons.

Being the most appropriate procedure with a dummy-coded outcome, binomial logistic regression analyses were used to examine the third research question with any cognitive impairment (yes = 1/no = 0) or type of transplant (living donor = 1/deceased donor = 2) as outcome. Furthermore, we calculated linear regression models to examine associations with creatinine and creatinine clearance, as well as time on dialysis, as outcomes.

## Results

### Participants

Fifty-nine patients (43 men, 16 women) with a mean age of 55 ± 13 years (min 22 years, max 76 years) completed the protocol. Thirty-four patients received a kidney graft from a living donor, and 25 from a deceased donor. On average, time since transplantation was 7 years (± 6yrs 10 months; min 3 months, max 29 years), and patients had been on dialysis 3 years and 10 months (± 3yrs 9 months; min 0 months, max 14 years) before transplantation. All of the patients had been on a calcineurin inhibitor (95% tacrolimus, 5% cyclosporine) since transplantation. Mean Mini-Mental Status Examination was 28.93 (± 1.23). There were no missing data in the variables included in the analyses.

#### Proportion and severity of cognitive impairment

**Fig. 1:**
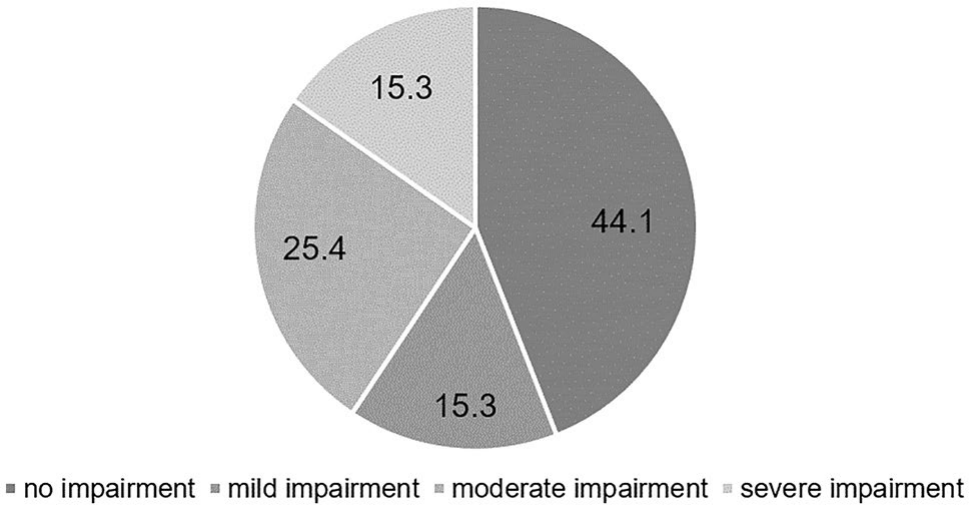
Proportion of patients per degree of cognitive impairment

Twenty-six patients did not show any cognitive impairment, 9 were mildly impaired, 15 moderately impaired, and 9 severely impaired as defined by our criteria above (see Fig. 1).

For descriptive values of socioeconomic and medical variables and associations with the degree of cognitive impairment see Tables 1 and 2.

**Table 1:** Means (M) and standard deviations (SD) of demographic and medical variables, and influence of these variables on cognitive impairment severity (ordinal logistic regressions)

	No impairment (n = 26)	Mild impairment (n = 9)	Moderate impairment (n = 15)	Severe impairment (n = 9)	*p*-value
	*M(SD)*	*M(SD)*	*M(SD)*	*M(SD)*	
Age in years	52 (13)	58 (12)	53 (13)	64 (9)	.055
Age at tx in years	46 (14)	54 (12)	46 (15)	52 (16)	.374
**Time since tx in months**	**73 (65)**	**57 (35)**	**79 (75)**	**156 (130)**	**.020**
Time on dialysis until tx in months	41 (42)	64 (53)	40 (47)	54 (48)	.594
Education years	14 (2)	14 (3)	12 (4)	13 (1)	.174
HADS depression score	5.12 (4.34)	3.11 (4.14)	5.60 (3.79)	5.00 (2.65)	.920
Systolic blood pressure	114.46 (64.32)	138.67 (11.27)	141.87 (18.95)	105.44 (77.93)	.485
Diastolic blood pressure	66.08 (49.48)	82.00 (9.90)	84.20 (6.37)	54.44 (58.01)	.772
Pulse pressure	56.50 (46.56)	69.44 (5.57)	73.40 (11.16)	53.00 (57.73)	.459
Hemoglobin	124.65 (18.12)	136.56 (17.62)	130.67 (18.73)	129.00 (21.68)	.316
Albumin	46.20 (4.22)	45.11 (3.01)	45.61 (4.08)	44.11 (3.57)	.206
Bicarbonate	22.81 (1.79)	22.87 (1.51)	22.81 (2.32)	23.08 (1.49)	.790
Creatinine	1.88 (0.99)	1.61 (0.61)	1.65 (0.70)	2.16 (1.68)	.861
Creatinine clearance	50.75 (23.86)	68.43 (54.55)	57.92 (24.41)	49.75 (32.34)	.834

*tx* kidney transplantation, *HADS* hospital anxiety and depression scale

**Table 2:** Number of patients in each group and association of variables with impairment severity (ordinal logistic regressions)

		No impairment (*n* = 26)	Mild impairment (*n* = 9)	Moderate impairment (*n* = 15)	Severe impairment (*n* = 9)	*p*-value
What kind of transplant	19 living (73%)		3 living (33%)	9 living (60%)	3 living (33%)	.056
Sex	18 m (69%) 8f		7 m (78%) 2f	12 m (80%) 3f	6 m (67%) 3f	.761
Depression	4 (15%)		0 (0%)	1 (93%)	3 (33%)	.499
Arterial hypertension	24 (92%)		8 (89%)	15 (100%)	9 (100%)	.326
Coronary heart disease	5 (19%)		1 (11%)	5 (33%)	2 (22%)	.512
Diabetes mellitus	7 (27%)		2 (22%)	4 (27%)	4 (44%)	.473
TIA	1 (4%)		0 (0%)	2 (13%)	1 (11%)	.271
Absolute arrhythmia	2 (8%)		0 (0%)	2 (13%)	1 (11%)	.578
Smoking ever	7 (27%)		2 (22%)	7 (47%)	4 (44%)	.086
Cortisone	11 (42%)		5 (56%)	8 (53%)	3 (33%)	.968

*TIA* transient ischemic attack, *m* men, *f* women

#### Cognitive profile

**Fig.2:**
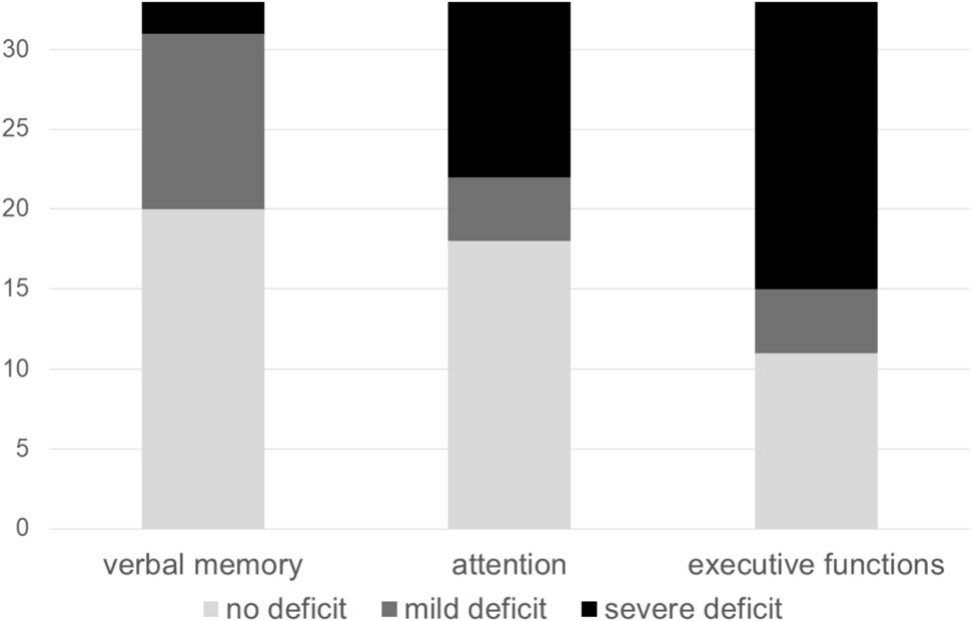
Number of patients with deficit per cognitive domain in patients with any cognitive impairment (*n* = 33)

In the sub-group of kidney transplant-recipients with any cognitive impairment (*n* = 33), there was a significant difference in deficit severity between verbal memory, attention, and executive functions (*χ*^*2*^*(2)* = 7.11*, p* = 0.029*),* see Fig. 2*.* From the three compared domains, cognitively impaired patients, on average, showed the lowest deficit in verbal memory, and the highest in executive functions (mean rank verbal memory deficit = 1.74, mean rank attention deficit = 1.97, mean rank executive function deficit = 2.29*).* However, neither the difference between deficit severity in verbal memory and deficit severity in attention (*z* = − 0.23*, adjusted p* = 1.00*)*, nor the difference between deficit severity in attention and deficit severity in executive function (*z* = − 0.32*, adjusted p* = 0.589*)* was statistically significant. The difference between deficit severity in verbal memory and deficit severity in executive functions was significant by trend (*z* = − 0.55*, adjusted p* = 0.080*, r* = 0.10*,* small effect)*.*

#### Differences in cognitive impairment between kidney transplant recipients after living and deceased donation

As can be seen in Table 1, an ordinal logistic regression with degree of cognitive impairment (no, mild, moderate, severe) as outcome and type of transplantation as predictor, showed a by-trend association between the two (*OR* = 0.39; 95% CI [0.148; 1.025]; *Wald χ*^*2*^_*(1)*_ = 3.65, *p* = 0.056). A binomial logistic regression using any cognitive impairment (yes/no) as outcome showed a significant effect: patients with a graft from a deceased donor were, on average, three times more likely to have any cognitive impairment than patients with a graft from a living donor (*OR* = 3.26, 95% CI [1.08,9.83], Wald *χ*^*2*^_*(1)*_ = 4.39, *p* = 0.036; model parameters: *χ*^*2*^_*(1)*_ = 4.65, *p* = 0.031; model explained 10% of variance (Nagelkerke R^2^) and correctly classified 63% of the cases). However, when controlled for the effect of age, the association just barely failed to reach statistical significance at the 0.05 level (*OR* = 3.03, 95% CI [0.99,9.32], Wald *χ*^*2*^_*(1)*_ = 3.74, *p* = 0.053; model parameters: *χ*^*2*^_*(1)*_ = 6.77, *p* = 0.034; model explained 15% of variance (Nagelkerke R^2^) and correctly classified 70% of the cases). In a next step, we also included time since transplantation, which was found to be significantly associated with degree of cognitive impairment in our prior analysis (see Table 1). This model explained 16% of variance (Nagelkerke R^2^) and correctly classified 68% of the cases. Again, type of transplant was only associated with cognitive impairment (yes/no) by trend in this model (*OR* = 2.86, 95% CI [0.92,8.90], Wald *χ*^*2*^_*(1)*_ = 3.30, *p* = 0.069; model parameters: *χ*^*2*^_*(1)*_ = 7.26, *p* = 0.064).

There was a significant association between the type of transplantation and time on dialysis: Patients who received a graft from a living donor had spent less time on dialysis than patients who received a graft from a deceased donor (*OR* = 1.04, 95% CI [1.02,1.06]; model parameters: *χ*^*2*^_*(1)*_ = 22.57, *p* < 0.001; model explained 43% of variance (Nagelkerke R^2^) and correctly classified 76% of the cases). However, although the cognitively unimpaired group, on average, spent less time on dialysis (no impairment: *M* = 40.61(± 42.38) months, impairment: *M* = 50.50(± 48.41) months), this effect was not statistically significant (*OR* = 1.00, 95% CI [0.99, 1.02]; model parameters: *χ*^*2*^*(1)* = 0.70, *p* = 0.404). Therefore, time on dialysis before transplantation does not explain the association between type of transplantation and cognitive impairment in our sample [24].

Similarly, the type of transplantation was significantly associated with creatinine levels: Patients who received a kidney graft from a living donor had lower creatinine levels than patients who received a kidney graft from a deceased donor (linear regression, *F*_*(1,57)*_ = 5.10, *β* = 0.29, *p* = 0.028, *R*^*2*^ = 0.80). There was, however, no association between creatinine levels and cognitive impairment (logistic regression, *OR* = 0.91, 95% CI [0.94, 1.51]; model parameters: *χ*^*2*^_*(1)*_ = 0.14, *p* = 0.709), which rules out current kidney function as a mediator between type of graft and cognitive impairment in our sample [24]. Type of transplantation did not predict creatinine clearance (*F*_*(1,45)*_ = 1.64, *β* = -0.19, *p* = 0.207), nor did creatinine clearance predict cognitive impairment (*OR* = 1.01, 95% CI [0.99,1.03]; model parameters: *χ*^*2*^_*(1)*_ = 0.70, *p* = 0.403).

## Discussion

We used a comprehensive neuropsychological test battery to examine the cognitive profile of patients who received a kidney graft in the past. In our study, we found a high proportion of cognitive impairment in this population. Overall, 55% suffered from any cognitive impairment (15% mild, 25% moderate and 15% severe). This is consistent with the proportion of cognitive impairment after kidney transplantation found in other studies [3]. On the other hand, it exceeds the prevalence of mild to severe cognitive impairment in the normal population [25]. Compared to persons with ESKD on dialysis, the rate of cognitive impairment is lower [1, 2] and less severe [2]. Meta-analytic data show that, after transplantation, patients have significantly better cognition than before transplantation [9]. Although cognition thus seems to improve after kidney transplantation, our data suggest that cognitive impairment in dialysis patients is not completely reversible.

In particular, while cognitively impaired patients, on average, showed only mild deficits in verbal and visual memory, they showed a clear deficit in executive functioning. This cognitive profile is similar to that of the population on dialysis, in which a significant impairment of executive function was also evident [1, 2, 26]. Comparing the domains, the difference between deficit in executive function and deficit in the other domains was only statistically significant by trend. This could be due to a restriction in statistical variance with first standardizing (norm values) and then categorizing (no, mild, and severe deficit) the raw values. This was, however, a necessity before entering the values into one comprehensive statistical model. Executive functioning is a complex ability, which is particularly reliant on the structural integrity of functional brain networks [27]. As such, subcortical white matter lesions, as seen in patients with CKD [28], could have an especially detrimental effect on this cognitive domain. Although former research found an increase in white matter volume in kidney transplant recipients after surgery, which corresponded with cognitive performance [29], this might not be able to completely reverse the effect on executive functioning. Studies are now needed that investigate whether the deficit in executive function can be targeted by cognitive training.

Additionally, we found that patients who received a kidney from a living donor were less likely to have a cognitive impairment than patients who received a kidney from a deceased donor. Interestingly, this effect was independent of time on dialysis before transplantation as well as of creatinine levels, and creatinine clearance. However, the association failed to reach statistical significance when considering age, and time since transplantation as confounders. Therefore, we have to interpret the finding with great caution. In studies with hemodialysis patients, it has been assumed that hemodynamic changes (changes in cerebral perfusion, low blood pressure) during dialysis are responsible for the impairment of cognitive function [15]. We might speculate that the white matter damage is caused by dialysis early on and does not increase with a longer dialysis duration. Another explanation is that cognitive impairment is reliant on other factors such as cardiovascular risk factors, which were found to differ between patients with living vs. deceased kidney donors after surgery [30]. More longitudinal multi-methodical research is clearly needed to investigate the relationship between dialysis, kidney transplantation, and cognitive impairment. The limited statistical robustness of the effect when entering control variables in the model (although still being significant by trend) is probably due to our small sample size and highlights the need to investigate this effect in a bigger sample.

Certain limitations of this study should be addressed in future research: In this part of the study, we included only patients who have already received a kidney graft. Also, the current analyses were cross-sectional in nature. Thus, no causality can be implied by our findings and we cannot draw conclusions on the development of the cognitive profile before and after transplantation. The latter is currently being investigated in another study population [16]. Furthermore, our study does not include brain imaging, which makes assumptions about the relationship between white matter damage and cognitive impairment in our sample merely speculative. Lastly, our sample consists of a rather small heterogeneous group of kidney graft recipients in terms of duration since transplantation and current health status. This might have led to larger statistical variance and ultimately smaller effect sizes, which could have been harder to detect.

On the other hand, our data rely on thorough and comprehensive neuropsychological testing which made it possible to compare different cognitive domains. Furthermore, thorough medical assessment provided us with the opportunity to investigate the influence of a multitude of possible causes leading to cognitive impairment in this patient group.

The present study may enhance our understanding of the nature of cognitive impairment in kidney transplant patients. Moreover, the results of our study have important implications for the prevention and treatment of cognitive impairment in kidney transplant recipients. By increasing knowledge on the neurocognitive profile, it is possible to create individualized training programs to positively impact cognitive deficits in these individuals.

## Data Availability

The datasets used or analyzed during the current study are available from the corresponding author on reasonable request.

## Code availability

No new code was produced in this study.

## References

[1] Murray AM et al (2006) Cognitive impairment in hemodialysis patients is common. Neurology 67(2):216–223 16864811 10.1212/01.wnl.0000225182.15532.40

[2] Karakizlis H et al (2022) Assessment of cognitive impairment and related risk factors in hemodialysis patients. J Nephrol 35(3):931–942 34655416 10.1007/s40620-021-01170-3 PMC8995241

[3] Gupta A et al (2017) Prevalence and correlates of cognitive impairment in kidney transplant recipients. BMC Nephrol 18(1):158 28499360 10.1186/s12882-017-0570-1 PMC5429555

[4] Ozcan H et al (2015) Kidney transplantation is superior to hemodialysis and peritoneal dialysis in terms of cognitive function, anxiety, and depression symptoms in chronic kidney disease. Transplant Proc 47(5):1348–1351 26093716 10.1016/j.transproceed.2015.04.032

[5] Griva K et al (2006) Cognitive functioning pre- to post-kidney transplantation–a prospective study. Nephrol Dial Transplant 21(11):3275–3282 16861731 10.1093/ndt/gfl385

[6] Van Sandwijk MS et al (2016) Cognitive changes in chronic kidney disease and after transplantation. Transplantation 100(4):734–742 26479287 10.1097/TP.0000000000000968

[7] Jurgensen A, Qannus AA, Gupta A (2020) Cognitive function in kidney transplantation. Curr Transplant Rep 7:145–153 32905482 10.1007/s40472-020-00284-0 PMC7473337

[8] Peng D, C.i.S.o.G. Geriatric Neurology Group, and C.P.G.f.C.I.o.C.S.V.D.W. Group (2019) Clinical practice guideline for cognitive impairment of cerebral small vessel disease. Aging Med (Milton) 2(2):64–73 31942514 10.1002/agm2.12073 PMC6880706

[9] Joshee P et al (2018) Meta-analysis of cognitive functioning in patients following kidney transplantation. Nephrol Dial Transplant 33(7):1268–1277 28992229 10.1093/ndt/gfx240 PMC6031036

[10] Patzer RE et al (2016) Medication understanding, non-adherence, and clinical outcomes among adult kidney transplant recipients. Clin Transplant 30(10):1294–1305 27447351 10.1111/ctr.12821 PMC5061615

[11] McAdams-DeMarco MA et al (2018) Dementia, Alzheimer’s disease, and mortality after Hemodialysis Initiation. Clin J Am Soc Nephrol 13(9):1339–1347 30093374 10.2215/CJN.10150917 PMC6140560

[12] Murray AM, Knopman DS (2010) Cognitive impairment in CKD: no longer an occult burden. Am J Kidney Dis 56(4):615–618 20851318 10.1053/j.ajkd.2010.08.003 PMC2943494

[13] Fakhr Yasseri AM et al (2021) Living versus deceased kidney transplantation: Comparison of complications. Urologia 88(3):185–189 33602045 10.1177/0391560321993540

[14] Weiner DE et al (2017) Cognitive function and kidney disease: baseline data from the systolic blood pressure intervention trial (SPRINT). Am J Kidney Dis 70(3):357–367 28606731 10.1053/j.ajkd.2017.04.021 PMC5572661

[15] Findlay MD et al (2019) Investigating the relationship between Cerebral blood flow and cognitive function in Hemodialysis Patients. J Am Soc Nephrol 30(1):147–158 30530658 10.1681/ASN.2018050462 PMC6317612

[16] Karakizlis H et al (2022) Neuropsychological Assessment of Cognitive Impairment in Kidney Transplantation (NAsKiT) and its related risk factors: a study protocol. J Nephrol 35(7):1933–1941 35763254 10.1007/s40620-022-01376-z PMC9458686

[17] Helmstaedter C, Lendt M, Lux S, Verbaler Lern- und Merkfähigkeitstest (VLMT) (2001) Göttingen: Beltz Test.

[18] Shin MS et al (2006) Clinical and empirical applications of the Rey-Osterrieth complex figure test. Nat Protoc 1(2):892–899 17406322 10.1038/nprot.2006.115

[19] Tombaugh TN (2004) Trail making Test A and B: normative data stratified by age and education. Arch Clin Neuropsychol 19(2):203–214 15010086 10.1016/S0887-6177(03)00039-8

[20] Härting C, Markowitsch HJ, Neufeld H, Calabrese P, Deisinger K, Kessler J (2000) Wechler Memory Scale- Revised, deutsche Version Göttingen: Hogrefe

[21] Folstein MF, Folstein SE, McHugh PR (1975) Mini-mental state A practical method for grading the cognitive state of patients for the clinician. J Psychiatr Res 12(3):189–198 1202204 10.1016/0022-3956(75)90026-6

[22] Petersen RC et al (1999) Mild cognitive impairment: clinical characterization and outcome. Arch Neurol 56(3):303–308 10190820 10.1001/archneur.56.3.303

[23] American-Psychiatric-Association (1987) Diagnostic and statistical manual of mental disorders, 3rd edn. American Psychiatric Press

[24] Baron RM, Kenny DA (1986) The moderator-mediator variable distinction in social psychological research: conceptual, strategic, and statistical considerations. J Pers Soc Psychol 51(6):1173–1182 3806354 10.1037//0022-3514.51.6.1173

[25] Dlugaj M et al (2010) Prevalence of mild cognitive impairment and its subtypes in the Heinz Nixdorf Recall study cohort. Dement Geriatr Cogn Disord 30(4):362–373 20956854 10.1159/000320988

[26] Drew DA, Weiner DE, Sarnak MJ (2019) Cognitive impairment in CKD: pathophysiology, management, and prevention. Am J Kidney Dis 74(6):782–790 31378643 10.1053/j.ajkd.2019.05.017 PMC7038648

[27] Coenen M et al (2023) Strategic white matter hyperintensity locations for cognitive impairment: A multicenter lesion-symptom mapping study in 3525 memory clinic patients. Alzheimers Dement 19(6):2420–2432 36504357 10.1002/alz.12827

[28] Wei CS et al (2022) Association between white matter hyperintensities and chronic kidney disease: a systematic review and meta-analysis. Front Med (Lausanne) 9:770184 35592851 10.3389/fmed.2022.770184 PMC9112853

[29] van Sandwijk MS et al (2020) Cognitive improvement after kidney transplantation is associated with structural and functional changes on MRI. Transplant Direct 6(3):e531 32195322 10.1097/TXD.0000000000000976 PMC7056275

[30] Yazbek DC et al (2012) Cardiovascular disease in early kidney transplantation: comparison between living and deceased donor recipients. Transplant Proc 44(10):3001–3006 23195014 10.1016/j.transproceed.2012.03.061

